# ICI 182,780 has agonistic effects and synergizes with estradiol-17 beta in fish liver, but not in testis

**DOI:** 10.1186/1477-7827-4-67

**Published:** 2006-12-27

**Authors:** Patrícia IS Pinto, Pratap B Singh, João B Condeça, Helena R Teodósio, Deborah M Power, Adelino VM Canário

**Affiliations:** 1 Centro de Ciências do Mar, CIMAR-Laboratório Associado, University of Algarve, Faro, Portugal; 2 Departmentof Zoology, T.D. College, Jaunpur-222002, India

## Abstract

**Background:**

ICI 182,780 (ICI) belongs to a new class of antiestrogens developed to be pure estrogen antagonists and, in addition to its therapeutic use, it has been used to knock-out estrogen and estrogen receptor (ER) actions in several mammalian species. In the present study, the effects and mechanism of action of ICI were investigated in the teleost fish, sea bream (Sparus auratus).

**Methods:**

Three independent in vivo experiments were performed in which mature male tilapia (Oreochromis mossambicus) or sea bream received intra-peritoneal implants containing estradiol-17 beta (E2), ICI or a combination of both compounds. The effects of E2 and ICI on plasma calcium levels were measured and hepatic and testicular gene expression of the three ER subtypes, ER alpha, ER beta a and ER beta b, and the estrogen-responsive genes, vitellogenin II and choriogenin L, were analyzed by semi-quantitative RT-PCR in sea bream.

**Results:**

E2 treatment caused an increase in calcium levels in tilapia, while ICI alone had no noticeable effect, as expected. However, pretreatment with ICI synergistically potentiated the effect of E2 on plasma calcium in both species. ICI mimicked some E2 actions in gene expression in sea bream liver upregulating ER alpha, vitellogenin II and choriogenin L, although, unlike E2, it did not downregulate ER beta a and ER beta b. In contrast, no effects of E2 or ICI alone were detected in the expression of ERs in testis, while vitellogenin II and choriogenin L were upregulated by E2 but not ICI. Finally, pretreatment with ICI had a synergistic effect on the hepatic E2 down-regulation of ER beta b, but apparently blocked the ER alpha up-regulation by E2.

**Conclusion:**

These results demonstrate that ICI has agonistic effects on several typical estrogenic responses in fish, but its actions are tissue-specific. The mechanisms for the ICI agonistic activity are still unknown; although the ICI induced up-regulation of ER alpha mRNA could be one of the factors contributing to the cellular response.

## Background

Most estrogen actions are mediated by specific nuclear estrogen receptors (ERs), which classically regulate transcription by binding as dimers to specific estrogen-response elements (ERE) found in promoters of estrogen-responsive genes (ERGs) [1]. This transcriptional activity is dependent on conformational changes of the ERs two activation functions (AF): the N-terminal (ligand-independent) AF-1 and the C-terminal (ligand-dependent) AF-2, which function independently or synergistically to recruit and interact with coregulator (coactivator or corepressor) proteins leading to changes in the rate of gene transcription [2]. Two ER subtypes (α and β) are present in most vertebrates, although in teleost fish one ERα and two ERβ genes (βa and βb) have been identified [e.g. [3-5]].

There is also substantial evidence that estrogens also function via non-classical mechanisms [1]. These include indirect transcriptional activation through interaction of ligand-bound ER with other transcription factors, ER ligand-independent activation in response to intracellular signaling cascades, and rapid non-genomic actions initiated at the plasma membrane. However, it is not clear if these are mediated by a subset of nuclear ERs that localize to the plasma membrane, or by novel membrane receptors unrelated to ER or through both [1,6,7].

ERs are known to accept a wide range of ligands, including natural estrogens, synthetic estrogens or antiestrogens, phytoestrogens and a variety of xenoestrogens [8]. While many behave as estrogen agonists, other compounds may act either as agonists or antagonists depending on the species, tissue, promoter or ER subtype (the selective ER modulators, SERMs; e.g. tamoxifen) [9]. The tissue-selective effects of SERMs have been exploited to develop new drugs for the treatment of estrogen-related diseases, although some have unwanted side effects or generate resistance to treatment, in part due to their agonist effects in some tissues [10].

ICI 182,780 (trade names Faslodex, Fluvestrant) belongs to a new class of antiestrogens developed to have no agonistic effects, and besides its therapeutic potential demonstrated in clinical trials, it has been used as an alternative and efficient means to "knock-out" ER effects in studies of estrogen functions, and to establish the contribution of nuclear ERs to particular estrogen actions [11-14]. In mammals, ICI 182,780 appears to act at several levels to block estrogen actions [reviewed by [11,15]], but little is known about its effects and mechanisms of action in fish. ICI blocked E_2_-induced interstitial cell proliferation in immature rainbow trout (*Oncorhynchus mykiss*) testis [16] and the production of zona radiata proteins and vitellogenin in Atlantic salmon (*Salmo salar*) hepatocytes [17] and of vitellogenin in channel catfish (*Ictalurus punctatus*) [18] and Siberian sturgeon (*Acipenser baerii*) [19] hepatocytes. Agonistic actions have been identified in Atlantic croaker (*Micropogonias undulatus*), in which both estradiol-17β (E_2_) and ICI decreased gonadotropin-stimulated 11-ketotestosterone production in testicular fragments *in vitro*, although these rapid effects appeared to be mediated by interaction with membrane-bound receptors [20,21].

The objective of this study was to investigate the effects and mechanisms of action of ICI 182,780 (ICI) on several typical *in vivo *estrogenic responses in the teleost fish sea bream (*Sparus auratus*), in order to evaluate the potential of ICI as an agent to knock-out estrogen effects in fish. In a preliminary experiment with the more readily available tilapia (*Oreochromis mossambicus*) we established that ICI potentiated the calciotropic effect of E_2 _[22-24]. The effects of E_2 _and ICI on plasma calcium and on hepatic and testicular gene expression of the three ER subtypes and the estrogen-responsive genes vitellogenin II and choriogenin L (egg yolk and eggshell precursors, respectively) were then analyzed in sea bream, for which these molecular markers were available [5,25].

## Methods

### Fish

All animal maintenance and handling procedures were carried out in compliance with the recommendations of the Association of Animal Behavior [26]. Adult tilapia were obtained from a stock raised from fertilized eggs at the University of Algarve, Faro, Portugal, and maintained in 150L closed circuit freshwater aquaria at a water temperature of 24°C and 12L:12D (light-dark) photoperiod. Adult sea bream were obtained from TIMAR Cultura de Águas (Olhão, Portugal) and maintained at the University of Algarve in through-flow 500L seawater tanks with a water temperature of 17–21°C, 36‰ salinity and 12L:12D photoperiod. At least one weak before the start of each experiment, fish were randomly distributed between different tanks (one per treatment) and left to acclimatize during this period.

### Treatments and sampling

Three independent experiments were performed (see additional file 1 for a representation of the experimental design of the three experiments), in which anaesthetized tilapia or sea bream (2-phenoxyethanol, Sigma-Aldrich, Madrid, Spain, diluted 1:10,000 in seawater) received intra-peritoneal (i.p.) implants of coconut oil (200 μl/100 g body weight, Sigma-Aldrich) containing different doses of E_2 _(Sigma-Aldrich) and/or of ICI 182,780 (Tocris, Cookson Ltd, Bristol, UK). Fish were returned to their tanks and left undisturbed during the experimental period (1–11 days), during which they were fed daily at the normal rate.

The first experiment, with male tilapia, was designed to test the broad effects of ICI on plasma calcium, the concentration of which typically increases in response to estrogen [23,24]. Tilapia was chosen because we can easily obtain reproductively mature individuals all year round. The experiment was carried out in June/July with five groups, each composed of five sexually mature male tilapia (body weight 45.9 ± 3.6 g). The effect of ICI alone compared to control fish was tested in two groups in which i.p. implants of coconut oil containing 35 mg/kg body weight (bw) (I35 group) or the vehicle coconut oil alone (CTL, control group) were administered. Since it was expected that ICI would act as an E_2 _antagonist, we hypothesized that pre-treatment with ICI should inhibit the response to E_2 _including a lower calciotropic response to E_2_. To test this hypothesis three further groups of fish were pre-treated with either coconut oil vehicle, 35 mg/kg bw ICI or 10 mg/kg bw ICI in coconut oil, followed 3 days later by 10 mg/kg bw E_2 _in coconut oil (groups E, I35E3d and I10E3d, respectively). The CTL and I35 groups were also injected at this time with coconut oil alone. At each sampling point (days 3, 5 and 11), fish were anesthetized and blood samples collected from the caudal vein with heparinized (150 U/ml ammonium heparin, Sigma-Aldrich) 1 ml syringes. Plasma samples were obtained by centrifugation of whole blood (10,000 rpm for 5 min) and were stored at -20°C until the determination of plasma calcium levels.

Since the tilapia experiment demonstrated a significant agonistic effect of ICI, we have carried out an experiment in sea bream (sea bream experiment 1, see additional file 1 for a representation of the experimental design) to compare the gene expression in response to ICI or E_2 _at the dosage levels used in tilapia. The sea bream was chosen because we have available a range of molecular markers for estrogen responsive genes [5,25]. The experiment was carried out in September, at the beginning of spermiation, with three groups of eight mature male sea bream (body weight 367.1 ± 8.9 g) which received i.p. implants of coconut oil containing 10 mg/kg bw E_2 _(E group), 10 mg/kg bw ICI (I group) or coconut oil alone (CTL, control group). Twenty eight hours later, fish were over-anesthetized in 1:5,000 2-phenoxyethanol:seawater, killed by decapitation and transverse sections of testis and liver were collected, snap frozen in liquid nitrogen and stored at -80°C for subsequent RNA extraction.

In the third experiment, carried out in November (sea bream experiment 2, see additional file 1 for a representation of the experimental design), we set out to examine the effect of lower, more physiologically relevant, dosages of ICI and E_2_, separately or in combination, on plasma calcium and gene expression in sea bream. For that purpose six groups of eight mature spermiating male sea bream (body weight 239.2 ± 3.3 g) were injected with the vehicle (coconut oil) in the first day, except for group IE3d which was injected with 4 mg/kg bw ICI in coconut oil. After three days, fish were injected with coconut oil implants containing 1 mg/kg bw E_2 _(E1 group), 0.1 mg/kg bw E_2 _(E0.1 group), 4 mg/kg bw ICI (I group), 4 mg/kg bw ICI plus 1 mg/kg bw E_2 _(IE group) or 1 mg/kg bw E_2 _(administrated to the IE3d group, injected with ICI in the first day), while the control group (CTL) was injected with coconut oil alone. Forty eight hours later, blood was collected and plasma samples recovered as described above and stored at -20°C until used for determination of calcium and E_2 _(see below). Fish were killed by decapitation and transverse sections of liver and testis sampled, frozen in liquid nitrogen and stored at -80°C for subsequent RNA extraction.

### Quantification of E_2 _and calcium plasma levels

Total plasma calcium (bound plus free) was measured in duplicate in 10 μl plasma samples from individual fish using a colorimetric assay (Calcium kit, procedure no.587, Sigma-Aldrich). E_2 _was quantified in individual plasma samples by radioimmunoassay using specific antiserum (Research Diagnostics, Flanders, New Jersey, USA) as described in Guerreiro *et al*. [27]. The cross-reaction of E_2 _antisera with ICI was approximately 20% in the middle of the linear portion of the standard curve but there was no parallelism with the standard curve of the assay.

### Semi-quantitative RT-PCR

Total RNA from male sea bream liver or testis was extracted from frozen tissues using TRI Reagent (Sigma-Aldrich) and cDNA was reverse transcribed from 4 μg of total RNA using random primers and M-MLV reverse transcriptase (Invitrogen, Carlsbad, CA, USA) in a 30 μl reaction. The mRNA expression of sea bream ERs, vitellogenin II (VgII), choriogenin L (ChgL) and the internal control gene coding for 18S ribosomal RNA (18S) was analyzed by semi-quantitative RT-PCR using the same reaction conditions, primers and annealing temperatures previously described by Pinto *et al*. [5,25]. These parameters are represented in Table 1, together with the cycle numbers optimized for the detection of each gene in the exponential phase of amplification for each experiment/tissue analyzed in this study. No genomic DNA or cross-annealing with genes from the same family was detected with these primers [5,25]. Band intensities of RT-PCR products were quantified by densitometry as previously described [25] and relative expression values calculated as base ten logarithms of the expression ratios between each gene and 18S.

**Table 1:** Primers used for gene expression analysis by RT-PCR

				**Cycle number(N)**		
				
				**Exp1**		**Exp2**
				
**Gene product**	**Primer Sequence**	**bp**	**Ta (°C)**	**Liv**	**Tes**	**Liv**
**Estrogen receptor α **(ERα, AJ006039)	5'-CCCATCCAGTCAGCATTCA-3' 5'-TTGTCACGCCGCAGAACG-3'	374	57	25	25	25
**Estrogen receptor βa **(ERβa, AF136980)	5'-GCTGATGATCGGACTGATGTG-3' 5'-GGTGTACTGTTGGCGGAAAG-3'	348	59	27	23	29
**Estrogen receptor βb **(ERβb, AJ580049)	5'-TGATGATGTCACTCACCAACC-3' 5'-TTCAGCTCACGAAACCGA-3'	291	54	25	22	26
**Choriogenin L **(ChgL, CX734876)	5'-AGAGGGATGCTGTCGTAG-3' 5'-GTGATGCCTTTGGTAGTG-3'	290	56	25	30	18
**Vitellogenin II **(VgII, CX734956)	5'-CACTTGGCATTGGTCTCCC-3' 5'-ATGGTGCACTCAGCTGCATG-3'	130	58	20	25	18
**18S ribosomal RNA **(18S)	5'-TCAAGAACGAAAGTCGGAGG-3' 5'-GGACATCTAAGGGCATCACA-3'	495	59	18	18	15

Gene name abbreviation and accession number, primer sequence (forward and reverse, respectively), amplicon length (bp), annealing temperature (Ta) and optimized cycle numbers (N) for liver (Liv) or testis (Tes) cDNAs from Experiments (Exp) 1 and 2.

### Statistical analysis

Plasma calcium concentrations in the tilapia time-course experiment were log transformed and analyzed by two-way repeated-measures ANOVA, followed by a post-hoc Tukey test for pairwise multiple comparison. Calcium and E_2 _concentrations and semi-quantitative RT-PCR data (all log transformed) in the sea bream experiments were analyzed by one-way ANOVA followed by the Tukey test. Pearson correlations of expression levels among genes were calculated and probabilities determined with Bonferroni corrections. The software used in the analysis was SigmaStat v.3.00 (SPSS Inc, Chicago, USA). Data is presented as mean ± standard error of the mean and statistical significance was established at P < 0.05.

## Results

### Effect of E_2 _and ICI 182,780 on plasma calcium in tilapia

**Figure 1:**
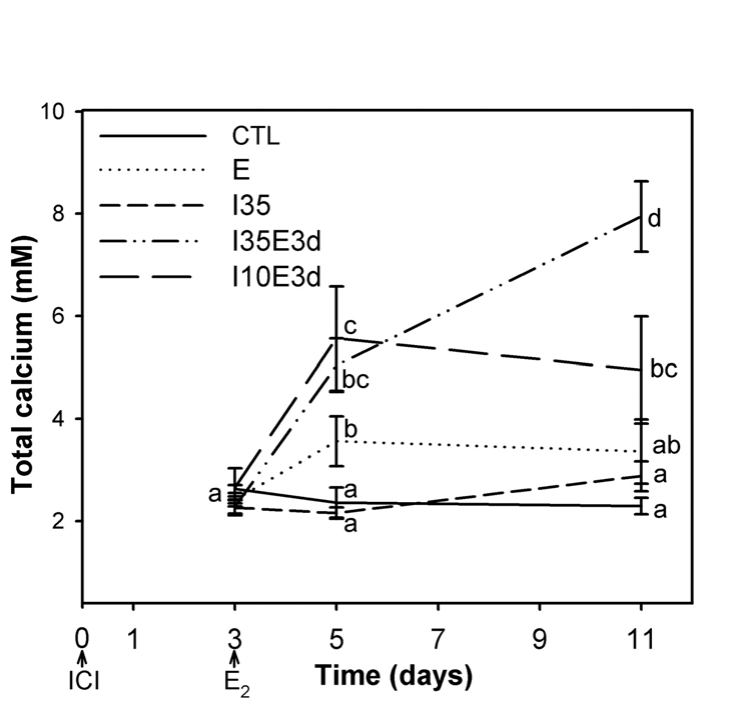
**Time-course of total calcium plasma response in tilapia**. Adult male tilapia received coconut oil implants with different combinations of estradiol (E_2_) and of the antiestrogen ICI 182,780 (ICI). Times of injection are represented by arrows on the lower panel. CTL = control group, coconut oil only; E = 10 mg/kg body weight E_2_; I_35 _= 35 mg/kg ICI; I_35_E3d and I_10_E3d = 10 mg/kg E_2 _injected three days after injection with 35 or 10 mg/kg ICI, respectively. Blood samples were collected 3, 5 and 11 days after the first injection. Different letters indicate statistically significant differences (P < 0.05) among treatments and sampling times, evaluated by two-way repeated-measures ANOVA using log_10 _of calcium plasma levels.

As expected, after 2 days of E_2 _treatment tilapia plasma calcium levels increased to almost twice those of the controls (Figure 1). In contrast, there were no statistically significant differences in calcium levels between control and ICI only-treated tilapia. However, ICI-pretreatment at the two doses tested strongly potentiated the effect of E_2 _on calcium causing a highly significant ~2 fold increase within two days and a further ~1.5 fold increase in the next 6 days with the highest ICI dose (I_35_E3d).

### Calcium and gene expression response to E_2 _and/or ICI 182,780 treatment in sea bream

**Figure 2:**
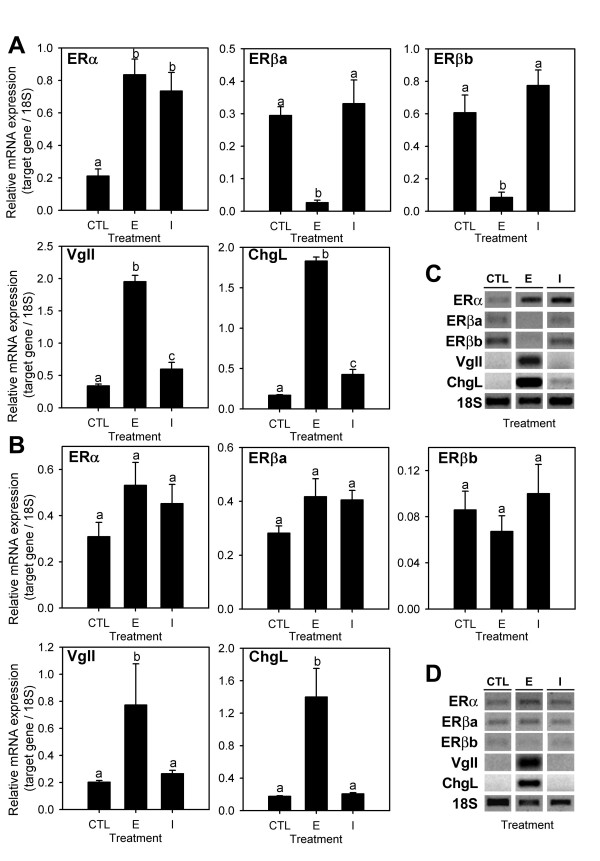
**Gene expression in E_2_-treated (high dose) sea bream**. Sea bream males received coconut oil implants with 10 mg/kg body weight E_2 _(E), 10 mg/kg ICI (I) or coconut oil alone (CTL) for 28 h. Liver (**A **and **C**) and testis (**B **and **D**) were analyzed for gene expression of estrogen receptors (ERα, ERβa, ERβb) and estrogen-responsive genes, vitellogenin (VgII) and choriogenin (ChgL) by semi-quantitative RT-PCR. Each bar (**A **and **B**) is the mean ± S.E.M. of the relative expression values (target gene/18S) of eight fish. Different letters indicate statistically significant differences between treatments (general linear model using log_10 _of the relative expression values, P < 0.05). The gel images (**C **and **D**) are representative RT-PCR products for each experimental group.

Since in the first experiment conducted in tilapia 10 and 35 mg ICI provoked a rise in calcium of similar magnitude after three days of E_2 _exposure, 10 mg/kg ICI and 10 mg/kg E_2 _treatments were used to investigate their short-term effects (28 h) on gene expression in sea bream. As expected, E_2 _treatment caused a significant up-regulation in expression of ERα (approx. 4-fold), VgII (6-fold) and ChgL (11-fold) in liver (Figure 2A). In contrast the mRNA levels of both ERβ subtypes significantly decreased approx. 10-fold compared to the control group. Interestingly, treatment with ICI also caused a significant up-regulation in ERα, VgII and ChgL, which was of the same magnitude to that of E_2 _treatment in the case of ERα but significantly less in the case of VgII and ChgL (1.8- and 2.5-fold increase, respectively, compared to control). ICI treatment had no effect on the expression of ERβa or βb. Strong positive Pearson correlations were found between the ERα, VgII and ChL responses to E_2 _and ICI in this tissue (Pearson coefficient 0.658–0.939, P < 0.01), but not between ERβa and ERβb.

In the testis, no statistically significant changes in gene expression were obtained for any of the ER subtypes (Figure 2B), while the expression levels of both VgII and ChgL were increased by E_2 _but remained unaltered by ICI treatment. In contrast to what was observed in the liver, ERα was positively correlated with ERβa (Pearson coefficient 0.640, P < 0.05) but not with VgII and ChgL, which were highly correlated among them (0.853, P < 0.001).

In order to further investigate and confirm the obtained effects of E_2 _and ICI on the different types of estrogenic response, a second sea bream experiment was performed in which lower doses of hormones were used (1 or 0.1 mg/kg E_2_, 4 mg/kg ICI) in different combinations and sampling conducted 48 h after E_2 _injection. Expression in testis was not analyzed due to the lack of response to ICI detected for ERs in sea bream experiment 1 and the high variability among individuals in the VgII and ChL response. Furthermore, the fish in the second sea bream experiment were at a stage of more advanced maturity (active spermiation) than in the first sea bream experiment (early spermiation) making direct comparisons difficult. In contrast, the liver showed more consistent and less variable results. To confirm the effectiveness of the treatments with the lower levels of hormones, E_2 _plasma levels were determined for each fish at the end of the experiment. Control sea bream had circulating plasma E_2 _levels of 0.6 ± 0.2 ng/ml, while in E_2_-implant groups these had increased 26-fold to 15.5 ± 4.0 ng/ml (E1 group) or 7-fold to 4.0 ± 0.6 ng/ml (E0.1). E_2 _plasma levels in the ICI-implanted groups (4.6 ± 1.8 ng/ml for I, 27.6 ± 7.9 for IE and 27.5 ± 7.0 for IE3d) were probably overestimated due to cross-reaction. The levels of E_2 _achieved with the implants are within the range observed in spawning sea bream [28]

**Figure 3:**
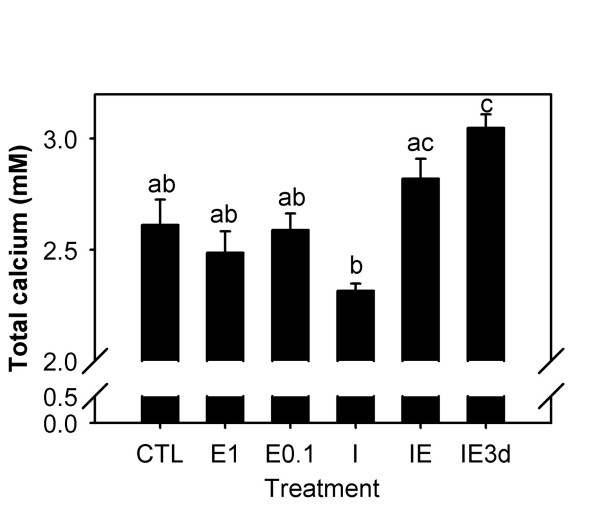
**Total calcium plasma levels in sea bream**. Sea bream males were sampled two days after receiving coconut oil implants containing 1 mg/kg body weight E_2 _(E_1_), 0.1 mg/kg E_2 _(E_0.1_), 4 mg/kg ICI (I), 4 mg/kg ICI plus 1 mg/kg E_2 _(IE), 1 mg/kg E_2 _in addition to 4 mg/kg ICI (IE3d) 3 days earlier, or coconut oil alone (CTL). Each bar is the mean ± S.E.M of the calcium plasma levels (mM) of eight fish. Different letters indicate statistically significant differences (P < 0.05) between treatments, evaluated by one-way ANOVA using log_10 _of calcium plasma levels.

As for calcium levels, no significant difference was detected between control fish and E_2 _at any dosage, ICI alone or the simultaneous treatment with E_2 _and ICI (Figure 3). However, in common with tilapia, pretreatment with ICI followed by E_2 _three days later (IE3d group) caused a statistically significant increase in the plasma calcium levels compared to control fish. A statistically significant difference between plasma calcium concentrations in ICI treatment only and the IE3d treatment group was also observed.

**Figure 4:**
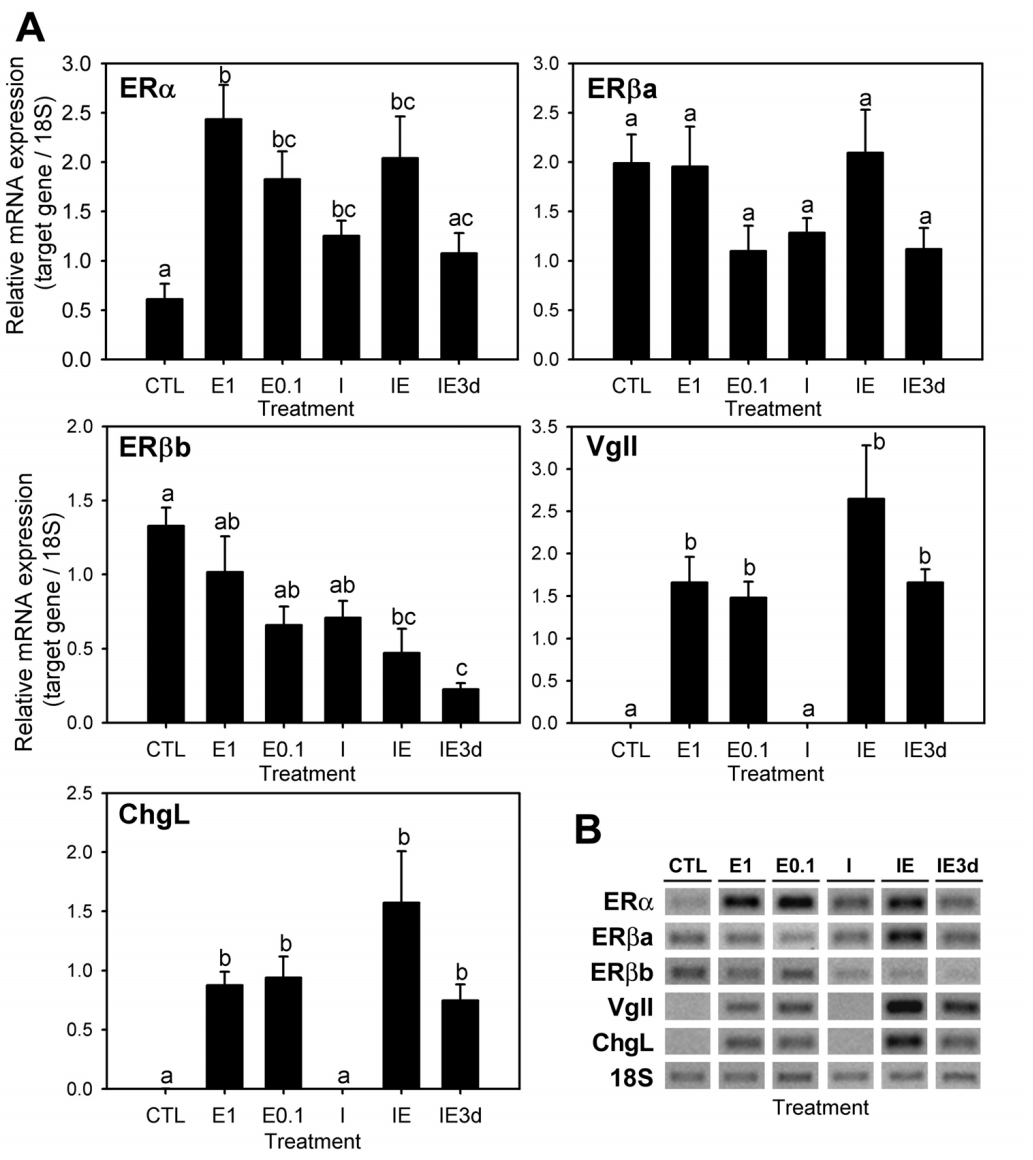
**Gene expression in E_2_-treated (low doses) sea bream**. Semi-quantitative RT-PCR of estrogen receptors (ERα, ERβa, ERβb) and estrogen-responsive genes, vitellogenin (VgII) and choriogenin (ChgL), in male sea bream liver (see Figure 3 for group abbreviations) 48 h following treatment. Each bar (**A**) is the mean ± S.E.M. of the relative expression values (target gene/18S) of eight fish. Different letters indicate statistically significant differences between treatments (general linear model using log_10 _of the relative expression values, P < 0.05). The gel images (**B**) are representative RT-PCR products for each experimental group.

As in the previous experiment, the hepatic expression levels of ERα were significantly increased by both doses of E_2 _and by ICI alone (Figure 4). Simultaneous administration of ICI and E_2 _(IE group) also caused a significant increase in the ERα expression levels, although it was not significantly different from that obtained with the same dose of E_2 _alone (E1). In contrast, in the group pretreated with ICI (IE3d group), ERα transcript levels were not significantly different from the control but differed from the E_2 _alone group (E1), suggesting an inhibition of the E_2_-induced ERα up-regulation.

For ERβa, no significant differences were detected between the different treatments, although a decrease compared to control was apparent for the lower dose of E_2 _(E0.1), ICI alone (I) and pretreatment with ICI followed by E_2 _(IE3d) (Figure 4). Similarly, with ERβb there was a trend for reduction in expression levels in response to all the treatments, with only the combination of E_2 _and ICI (IE and IE3d groups), significantly decreasing the expression level of this gene compared to the control, and with IE3d also decreasing it in comparison to the E_2_ only-treated groups (E1 and E0.1). The expression of both VgII and Chg was significantly increased by both doses of E_2 _and combined E_2_/ICI treatments, while no change in expression could be detected in the group treated with ICI alone. Strong positive Pearson correlations were found between the ERα, VgII and ChL response (Pearson coefficients 0.466–0.981, P < 0.01) and between ERβa and ERβb (Pearson coefficient 0.679, P = 0.00), while negative correlations were found between the response of ERβb and both VgII and ChgL (coefficients -0.435 and -0.398, respectively, P < 0.05).

## Discussion

This study demonstrated that, at least in fish, ICI does not always function as an anti-estrogen since it did not block the effects of an E_2 _challenge. Indeed, prior administration of ICI potentiated the response to E_2_. Furthermore, the agonistic response to ICI could also be detected at the level of gene expression and was different in liver and testis.

The level of total calcium in plasma is known to correlate with Vg protein and E_2 _plasma levels in females during vitellogenesis and in males in response to E_2 _exposure, and it is thus used as a vitellogenesis marker [22-24]. The lack of a statistically significant calcium elevation with E_2 _treatment alone in sea bream was probably due to the low doses and/or exposure time compared to previous sea bream experiments (>4 days, 10 mg/kg) [27] and to the tilapia experiment (>48 h, 10 mg/kg). ICI alone did not change total plasma calcium levels or may have slightly lowered calcium within 48 h (Figures 1 and 3), consistent with an antagonistic action. However, pretreatment with ICI synergistically potentiated the hypercalcemic effect of E_2 _in both sea bream and tilapia. This observation seems to indicate that the initial binding of ICI to ER effectively blocks ER binding to target genes (antiestrogenic action) in the liver, but subsequently E_2 _triggers a disproportionate agonistic response. Whether ICI acts by stimulating ER synthesis or at the level of ER responsiveness is not clear. In support for the first possibility is the fact that ERα levels in the liver of fish treated with ICI are upregulated and at similar levels to the E_2_-treated fish. However, it is surprising that ERα levels in E_2 _challenged fish after pretreatment with ICI are no higher than fish treated with ICI only (Figure 4). Analysis of the early time-course changes in this response is required to clarify the possible mechanism involved.

The ERα, VgII and ChgL up-regulation by E_2 _in liver is in accordance with our previous observations [25]. ERα autoregulation in liver is a common characteristic of oviparous animals [e.g. [29-32]] that has been attributed to ER involvement in the production of egg yolk and egg shell precursors vitellogenins and choriogenins, respectively, in the liver of mature females in response to E_2_. In contrast to ERα, the regulation of ERβ genes by estrogen is poorly investigated and appears to be much more variable, with these genes being either slightly up- or down-regulated depending on the species and ER subtype [33,34]. In this study, the expression of both ERβa and ERβb are strongly down-regulated by E_2 _in liver in the first sea bream experiment, and only slightly down-regulated in the second experiment in which lower doses are used, possibly indicating that their regulation is dose-dependent (and less sensitive to E_2 _than ERα). These results indicate a differential estrogen regulation of sea bream ERα and ERβ genes in liver and support the hypothesis that the role of the ERβ forms, in the transcriptional regulation of genes associated with reproduction in fish liver is probably less important than that of the ERα subtype and may depend on the life stage of the fish and/or the species. In contrast to the liver, a slight up-regulation by E_2 _of both ERα and ERβb but not ERβb is detected in testis, suggesting the regulation of ER subtypes varies among tissues, while the up-regulation of VgII and ChgL confirms its recent identification as ERGs in this tissue [25].

ICI mimicked the E_2 _effects in the liver, up-regulating ERα, VgII and ChgL in sea bream liver, but not in the testis and unlike E_2 _it did not down-regulate the two ERβ subtypes in liver, supporting tissue- and gene-specific effects for this compound. The simultaneous administration of ICI with E_2 _did not block the E_2 _effects on the expression of any of the genes, neither did the ICI pretreatment in the E_2_-induced up-regulation of ChgL and VgII, suggesting that ICI did not act as an antagonist. However, ICI pretreatment synergistically potentiated the E_2 _down-regulation of the ERβb gene, while it appeared to have an inhibitory effect on the E_2 _up-regulation of ERα (Figure 4), at least in the time-frame and doses analyzed (see above).

Taken together, these results contrast with the conventional classification of ICI as a "pure estrogen antagonist", which has been reported to block the effects of E_2 _and some partial agonists (e.g. tamoxifen) with no detected agonistic activities in several *in vivo *and *in vitro *models of estrogen action in different mammalian species [reviewed by [11,35]]. However, some recent *in vitro *studies have also reported agonistic or partial agonistic activities for ICI [36-40], which appear to depend on the species, the tissue, the ER subtype and the promoter, as reported for other SERMs.

The mechanisms in place for the agonistic effects in fish are as yet unknown. Most antiestrogens act through competitive binding to ERs and induction of an inactive conformation of the ligand-dependent AF-2 function of ERs, and their context-specific agonistic activities have been mainly attributed to a tissue- or promoter-specific activation of the ligand-independent AF-1 function or to the induction of a partially active AF-2 conformation [41]. ICI appears to act at several levels to completely block ER-mediated actions (better studied for the ERα subtype), including the competitive inhibition of agonist binding to ERα, the inhibition of ER dimerization, nuclear translocation and transcription activation through both AF-1 and AF-2, and increased ER protein degradation [reviewed by [11,15]]. While estrogens are known to rapidly down-regulate the ERα and ERβ protein levels in several mammalian cell types but up regulate its mRNA levels [42], ICI has been shown to cause ERα protein degradation without affecting the ERα mRNA levels [11], thereby leading to an effective reduction of the ER protein levels. Possible explanations for the partial ICI agonism are: 1) lack of ERα protein down-regulation, as observed in cells of the sheep uterus or in human breast cancer cells; 2) species-specific differences in the N- or C-terminal regions of ERs, which could influence ligand discrimination; 3) ER activation via non-classical mechanisms (e.g. non-genomic actions and indirect activation at AP-1 promoters); 4) ICI activation of other ER subtypes (nuclear ERβ or membrane ERs) or ER variant proteins whose relative expression depends on the cell type or species [36-40]. In addition, it was recently reported that ICI was able to promote human ERα interaction with the CBP/p300 but not the p160 family of coactivators in HeLa cells, although this was insufficient to promote transcription from the pS2 (an ERG) promoter [43].

In fish, unlike in mammals, estrogens have been shown to increase both ERα mRNA (through increased transcription and enhanced stability) and ER protein levels in liver [31,44,45]. The ICI up-regulation of ERα in liver detected in the present study could contribute to the observed agonistic effects, and the potentiation effects observed for the ICI pretreatment may be due to an increased responsiveness of the tissue at the time of E_2 _administration through sbERα up-regulation by ICI. Whether the ERα mRNA ICI up-regulation is followed by an increase in ERα protein level in liver, as occurs with E_2_, must be investigated in future studies.

The inability of ICI to inhibit the up-regulation of ERα, VgII and ChgL by E_2 _in fish liver (Figure 4) could also be interpreted as evidence for an ER-independent mechanism, as suggested for other ICI-insensitive actions [e.g. [46]]. However, this appears not to be the case, since ICI alone was able to up-regulate these genes (Figure 2) and because the E_2_-induced transcriptional activation of both ERα and VgII genes have been demonstrated to involve binding of the ER proteins to specific response-elements in their promoters [33,47,48], while the stabilization of their mRNAs has also been shown to be mediated by E_2_/ER complexes [44]. The dependence on ERα has also been demonstrated in some studies reporting ICI agonism in mammals by using ER-specific siRNA [39].

## Conclusion

In conclusion, at least in fish, ICI does not always function as an antiestrogen since it did not block the effects of an E_2 _challenge on several typical estrogenic actions. Indeed, prior administration of ICI strongly potentiated the response of plasma calcium to E_2_. Furthermore, this agonistic response to ICI could be detected at the level of gene expression and was different in liver and testis. The strong up-regulation of ERα in correlation with Vg and ChgL and the down-regulation of both sbERβs confirm that ERα is probably the most important ER subtype controlling liver gene expression in response to E_2_.

The identified agonistic effects suggest caution in the use of ICI as a pure antiestrogen to "knock-out" estrogen functions in fish, at least until their effects and mechanisms of action are better characterized. It would be also interesting to investigate the effects of other "pure antagonists" such as ICI 164,384 and RU 58668 on the estrogenic actions analyzed in this study.

## Competing interests

The author(s) declare that they have no competing interests.

## Authors' contributions

PISP planned and carried out the sea bream experiments, some of the RT-PCRs, gene expression quantification, statistical analysis and discussion of results, and wrote the manuscript. PBS and JBC planned and carried out the tilapia experiments and calcium measurements. HRT carried out RNA extractions, RT-PCRs and calcium in the sea bream experiments. DMP participated in the discussion of results and wrote the manuscript. AVMC devised the study, participated in the planning of all experiments, statistical analysis and discussion of results, and wrote the manuscript. All authors read and approved the final manuscript.

## Supplementary Material

Additional File 1 Experimental design of the three *in vivo *experiments performed. This table represents the dosages (mg hormone/kg body weight), treatment times and sampling times (●) used for the different experimental groups in each of the three experiments performed (A, tilapia experiment; B, sea bream experiment 1; and C, sea bream experiment 2). c.o. coconut oil. Click here for file
