# Supplementary material for: ICI 182,780 has agonistic effects and synergizes with estradiol-17 beta in fish liver, but not in testis
Source: Reprod Biol Endocrinol. 2006 Dec 27;4:67. doi: 10.1186/1477-7827-4-67 (PMC1769500; doi:10.1186/1477-7827-4-67)
Supplement: Additional File 1 — Experimental design of the three in vivo experiments performed. This table represents the dosages (mg hormone/kg body weight), treatment times and sampling times (●) used for the different experimental groups in each of the three experiments performed (A, tilapia experiment; B, sea bream experiment 1; and C, sea bream experiment 2). c.o. coconut oil. [file 1477-7827-4-67-S1.pdf]

A- Tilapia Experiment

Groups	Day 0	Day 3	5	11
**C**	c.o.	c.o.		
**E**	c.o.	10mg/kg E_2		
**I35**	35mg/kg ICI	c.o.		
**I35E3d**	35mg/kg ICI	10mg/kg E_2		
**I10E3d**	10mg/kg ICI	10mg/kg E_2		
**Sampling**		●	●	●

B- Sea bream experiment 1

Groups	Day 0	Day 1
**C**	c.o.	
**E**	10mg/kg E_2	
**I**	10mg/kg ICI	
**Sampling**		●

C- Sea bream experiment 2

Groups	Day 0	Day 3	5
**C**	c.o.	c.o.	
**E1**	c.o.	1mg/kg E_2	
**E0.1**	c.o.	0.1mg/kg E_2	
**I**	c.o.	4mg/kg ICI	
**IE**	c.o.	4mg/kgICI+ 1mg/kg E_2	
**IE3d**	4mg/kg ICI	1mg/kg E_2	
**Sampling**			●
